# Nintedanib Alleviates Experimental Colitis by Inhibiting CEBPB/PCK1 and CEBPB/EFNA1 Pathways

**DOI:** 10.3389/fphar.2022.904420

**Published:** 2022-07-14

**Authors:** Hailong Li, Jinhe Li, Ting Xiao, Yayue Hu, Ying Yang, Xiaoting Gu, Ge Jin, Hailong Cao, Honggang Zhou, Cheng Yang

**Affiliations:** ^1^ The State Key Laboratory of Medicinal Chemical Biology, College of Pharmacy and Key Laboratory of Molecular Drug Research, Nankai University, Tianjin, China; ^2^ High-throughput Molecular Drug Screening Centre, Tianjin International Joint Academy of Biomedicine, Tianjin, China; ^3^ Department of Gastroenterology and Hepatology, General Hospital, Tianjin Medical University, Tianjin Institute of Digestive Diseases, Tianjin Key Laboratory of Digestive Diseases, Tianjin, China

**Keywords:** super-enhancer, inflammatory bowel disease, nintedanib, CEBPB/PCK1, CEBPB/EFNA1

## Abstract

The super-enhancer, a cluster of enhancers with strong transcriptional activity, has become one of the most interesting topics in recent years. This study aimed to investigate pathogenic super-enhancer–driven genes in IBD and screen therapeutic drugs based on the results. In this study, through the analysis of differentially expressed genes in colitis patients from the GEO database and the analysis of the super-enhancer–associated database, we found that the super-enhancer pathogenic genes PCK1 and EFNA1 were simultaneously regulated by transcription factor CEBPB through two super-enhancers (sc-CHR20-57528535 and sc-CHR1-155093980). Silencing CEBPB could significantly inhibit the expression of PCK1 and EFNA1 and enhance the expression of epithelial barrier proteins claudin-1, occludin, and ZO-1. In LPS-induced Caco-2 cells, drugs commonly used in clinical colitis including tofacitinib, oxalazine, mesalazine, and sulfasalazine inhibited mRNA levels of CEBPB, PCK1, and EFNA1. In the drug screening, we found that nintedanib significantly inhibited the mRNA and protein levels of CEBPB, PCK1, and EFNA1. *In vivo* experiments, nintedanib significantly alleviated DSS-induced colitis in mice by inhibiting CEBPB/PCK1 and CEBPB/EFNA1 signaling pathways. At the genus level, nintedanib improved the composition of the gut microbiota in mice with DSS-induced experimental colitis. In conclusion, we found that PCK1 and EFNA1 are highly expressed in colitis and they are regulated by CEBPB through two super-enhancers, and we further demonstrate their role *in vivo* and *in vitro*. Nintedanib may be a potential treatment for IBD. Super-enhancers may be a new way to explore the pathogenesis of colitis.

## Introduction

Inflammatory bowel disease (IBD), a chronic nonspecific inflammatory bowel disease common in North America and Europe, includes ulcerative colitis and Crohn’s disease (Kaplan, 2015; Ng, 2015). In recent decades, IBD incidence has rapidly increased in newly industrialized regions such as Asia and has evolved into a global disease (Ng et al., 2013; Park et al., 2014). These conditions and high incidence rates have heavily burdened patients and the healthcare system (Kappelman et al., 2008; Kappelman et al., 2013). The cause of IBD is not clear. Environmental, infectious, immune, and genetic factors are closely related to the pathogenesis of IBD (Ramos and Papadakis, 2019). The traditional therapeutic drugs for IBD are mainly salicylic acid, steroid hormones, and immunosuppressant drugs, which have a limited remission rate and can cause serious side effects, including potential liver and kidney damage (Zoubek et al., 2019). Therefore, it is necessary to explore a more effective molecular mechanism and find suitable alternative drugs to treat IBD.

Enhancers, DNA sequences on the genome, precisely regulate the spatiotemporal expression of target genes through cis-interactions during cell development and differentiation (Witte et al., 2015; Li et al., 2021a). It is an important cis-regulatory element in the cell identity and the development process of multicellular organisms (Agrawal and Rao, 2021). Super-enhancers (SEs), a class of transcription enhancers newly discovered in recent years, usually contain the same components associated with enhancer activity, including transcription factors and cofactors (Hnisz et al., 2013; Whyte et al., 2013; Xi Wang et al., 2019). Compared with enhancers, super-enhancers can bind transcription factors at a higher density (Hnisz et al., 2013; Higashijima and Kanki, 2021). Therefore, super-enhancers can regulate higher levels of gene transcription.

It was found that super-enhancers not only determine the cell fate in the process of stem cell differentiation and development, but also play a key role in the occurrence and development of tumors, autoimmune diseases, and other diseases (Hnisz et al., 2013; Loven et al., 2013; Brown et al., 2014). For example, there are active super-enhancers near the oncogene c-MYC in pancreatic cancer and colorectal cancer, while no corresponding super-enhancers are found in the corresponding normal tissues (Hnisz et al., 2013). In common complex diseases such as systemic lupus erythematosus, rheumatoid arthritis, multiple sclerosis, and other immune-related diseases, it was found that the expression of pathogenic genes is highly correlated with the abnormal activation of some super-enhancers (Costa-Reis and Sullivan, 2013). Among the 72 single-nucleotide polymorphisms (SNPs) in systemic lupus erythematosus, 22 SNPs occurred in the super-enhancer region (Deng and Tsao, 2010).

Because super-enhancers play a key role in determining cell identity and development, we believe that super-enhancers are involved in the occurrence and development of IBD diseases. However, the role of pathogenic super-enhancers in the pathological process of IBD has not been reported. The purpose of this study is to predict the potential pathogenic super-enhancers of IBD. The study of the role of super-enhancers in the development of IBD will help us to further explore the pathogenesis of this refractory disease and find new therapeutic targets and candidate drugs.

## Materials and Method

### Identification of Differentially Expressed Genes

The GEO database is a database for storing chips, second-generation sequencing, and other high-throughput sequencing data. The GEO database was searched to find the datasets (GSE107499, GSE75214, and GSE59071) containing differential genes in colon tissues of normal people and IBD patients. The differentially expressed genes were analyzed and found by the GEO2R tool.

### Prediction of Pathogenic Genes Driven by Super-enhancers

SEA version 3.0 is an online database that can provide information about super-enhancers. By collecting and analyzing the public Chip-Seq data, the SEA website contains information on the super-enhancers and their related genes found in different cells and tissues. In SEA, we identified the super-enhancers in the human sigmoid colon using H3k27ac Chip-sequencing and collected the information on the super-enhancer, including super-enhancer ID, genomic site, length, related genes, and transcription factors. Then, the super-enhancer–related genes and differentially expressed genes were cross-linked to obtain the differentially expressed genes driven by the super-enhancers. The interaction network between transcription factors, super-enhancers, and related genes was constructed using Cytoscape software.

### KEGG Pathway Analysis

The overlapping genes were analyzed by the KEGG pathway. The KEGG pathway data were uploaded to the Hiplot website to form a bubble chart.

### LPS-Infected Caco-2 Cells

The Caco-2 cell line was purchased from Wuhan Procell Life Technology Co., Ltd (Wuhan, China) and cultured in DMEM high-glucose medium (Solarbio, China) supplemented with 20% fetal bovine serum (FBS, Yeasen, China). All of the media contained 50 U/mL penicillin and 50 U/mL streptomycin. All cells were incubated with 5% CO_2_ at 37°C. After pre-protecting the cells for 2 h by adding the drug or an equal volume of vehicle, cells were treated with 1 μg/ml LPS to stimulate Caco-2 cells for 24 h, and the protein and mRNA expression levels were detected.

### RNA Extraction and Quantitative Real-Time Polymerase Chain Reaction (qRT-PCR)

Total RNA was extracted from Caco-2 cells and colon tissues by TRIzol reagent (Tiangen, Beijing, China) and quantified by using a NanoDrop 8000 spectrophotometer (Thermo Fisher Scientific Inc., Waltham, MA, United States). RNA was reverse transcribed into cDNA with the first-strand cDNA synthesis Supermix kit (Yeasen, Shanghai, China), and the PCR system was prepared with the Yeasen qRT-PCR kit to amplify cDNA and performed with CFX96 touch (BIORAD, United States) according to the protocol of the manufacturer. The reaction conditions were as follows: predenaturation at 95°C for 30 s, denatured at 95°C for 10 s, annealed at 60°C for 20 s, extended at 70°C for 20 s, and repeated 40 cycles. GAPDH was used as the standard control. The forward and reverse primer sequences of CEBPB, PCK1, EFNA1, and GAPDH are as follows. The cycle threshold was automatically generated, and the relative expressions of CEBPB, PCK1, and EFNA1 mRNA were calculated by the 2^-△△Ct^ method.

For humans:

CEBPB forward primer: ACG​GGC​CGC​CCT​TAT​AAA​T; CEBPB reverse primer: CAGGCCACCAGGCGTTG; PCK1 forward primer: CGG​AAA​GAA​ACC​TGT​GGA​TCT​C; PCK1 reverse primer: CAG​ATG​TGG​ATG​TGA​TCA​GGC​T; EFNA1 forward primer: GCT​ATG​GAG​TTC​CTC​TGG​GC; EFNA1 reverse primer: ACG​TAG​TCA​TTC​AGC​TGC​ACA; GAPDH forward primer: GAC​AGT​CAG​CCG​CAT​CTT​CT; GAPDH reverse primer: GCG​CCC​AAT​ACG​ACC​AAA​TC;

For mice:

CEBPB forward primer: TTA​TAA​ACC​TCC​CGC​TCG​GC; CEBPB reverse primer: TTC​CAT​GGG​TCT​AAA​GGC​GG; PCK1 forward primer: ACA​CAC​GCA​AAC​TAC​CAA​GC; PCK1 reverse primer: GTC​CTC​GTA​ATG​TGG​GCA​GA; EFNA1 forward primer: GGA​AGA​ACA​AGG​AGT​GGA​GAC; EFNA1 reverse primer: CAG​GCA​GGG​TCA​ATA​ATG​GG; GAPDH forward primer: GGA​GAG​TGT​TTC​CTC​GTC​CC; GAPDH reverse primer: CCG​TTG​AAT​TTG​CCG​TGA​GT;

### Western Blot

After grouping Caco-2 cells and colon tissues, the RIPA lysate was used to extract the protein in the cells and tissues, and the protein content was measured using the BCA kit (Beyotime, Beijing), and 30 μg protein was taken for detection. The protein samples were added into polyacrylamide gel (8–15%) and then electrophoretic gel, then transferred to the PVDF membrane (Millipore, MA, United States), sealed for 2 h with 5% skim milk powder, and incubated overnight with 1–1,000 diluted antibody at 4°C. Subsequently, PVDF membranes were incubated with rabbit antibodies diluted 1:1 000 for 2 h, and protein bands were obtained after development using a biospectral gel imaging system (UVP, CA, United States). Finally, Image J software was used for gray analysis.

### Animal Experiment

Male C57BL/6J mice (SPF grade) aged 6–8 weeks, weighing 18–22 g, came from the animal experiment center of the Jinnan campus of Nankai University. Fifty SPF mice were randomly divided into normal group, dextran sodium sulfate (DSS) group, sulfasalazine (SASP) group, nintedanib low-dose group (Nin, 50 mg/kg), and nintedanib high-dose group (100 mg/kg), with 10 mice in each group. After adaptive feeding for 1 week, the mice in the normal group were free to eat and drink water, and for the mice in other groups, the daily drinking water volume of each mouse was calculated to be 6 ml, and the DSS solution was added to the daily drinking water volume for 8 consecutive days to establish the model of IBD in mice. While drinking 5% DSS solution daily, SASP (200 mg⋅kg^−1^⋅Day^−1^) and nintedanib (50 and 100 mg⋅kg^−1^⋅Day^−1^) were administered by gavage at 1–7 days, respectively. 0.5% CMC-Na was administered by gavage in the normal group and the DSS group for 7 days. The body weight, stool consistency (degree of diarrhea), and blood in stool were recorded every day. On the 8th day, after fasting for 12 h, 10% chloral hydrate was injected intraperitoneally. The colon and rectum of mice were washed with the PBS solution. 1 cm tissue from the anus was selected and placed in 10% formalin. The remaining intestinal tissue was put into a cryopreservation tube and stored at −80°C. The intestinal tissue was fixed in formalin for 24 h, dehydrated, embedded, sliced, stained with hematoxylin-eosin and Alcian blue (PH = 2.5, Beyotime, Beijing), and observed under the microscope.

### The Disease Activity Index Score

The characteristics of feces were observed, and the situation of fecal occult blood was determined and scored accordingly. DAI scoring criteria are as follows (Peng et al., 2019):

1) Body mass score: no change or increase, 0 point; reduce 1%–5%, 1 point; reduce 5%–10%, 2 points; reduce 10%–15%, 3 points; the reduction is greater than 15%, 4 points.
2) Fecal character score: normal, 0; soft stool and spherical stool, 1 point; paste or hemispherical stool and no anal attachment, 2 points; paste stool and anal adhesion, 3 points; loose stool, 4 points.
3) Fecal occult blood score: negative, 0; weak positive, 1 point; positive, 2 points; strong positive, 3 points; gross bloody stool, 4 points. DAI score = (body mass score + fecal character score + fecal occult blood score)/3.

### Pathological Staining

The sections were stained with hematoxylin-eosin and observed under the microscope. The histopathological evaluation criteria are as follows (Stillie and Stadnyk, 2009):

1) Degree of inflammation: none, mild, moderate, and severe;
2) Depth of lesion involvement: no lesion, mucosa, submucosa and transmural;
3) Recess destruction: no destruction, basal 1/3 destruction, basal 2/3 destruction, basal destruction, and only epithelial integrity;
4) The range of lesions involved: 1–25%, 26–50%, 51–75%, and 76–100%.

### Clinical Samples

A total of 3 ulcerative colitis tissues and 3 colonic ulcer distal control tissues were collected from healthy volunteers (1 male and 2 females, aged 49–63 years) and UC patients (2 males and 1 female, aged 37–58 years) during endoscopy at the Department of Digestive Diseases, Tianjin Medical University General Hospital (Tianjin, China). Patients with ulcerative colitis were untreated preoperatively. Samples were independently identified by two pathologists. These tissues were obtained from patients who underwent colonoscopy at Tianjin Medical University General Hospital after signing informed consent. The entire study was approved by the ethics committee of Tianjin Medical University General Hospital.

### Immunohistochemistry

The tissue sections were dewaxed, hydrated, and antigen repaired. After incubation with endogenous peroxidase blocker and immune serum (rabbit) with the immunohistochemical kit (Maixin, Fuzhou, China), the CEBPB Rabbit antibody (1: 50 dilution; Affinity) was incubated overnight at 4°C. The next day, nonspecific secondary antibody and streptavidin peroxidase were used for incubation, DAB (Maixin, Fuzhou, China) color development, hematoxylin staining, and the slices were sealed after dehydration. Finally Image J software was used for IHC scoring.

### Transfection siRNA

Caco-2 cells were inoculated into 96-well plates and cultured in a 5% CO_2_ incubator at 37°C for 24 h. When the cell density reached 50–70%, si-CEBPB (Gene Pharma, shanghai) and the negative control group (NC) were transfected into the cell line according to the instructions of the lipo 8000 reagents (Beyotime, Beijing). 48 h after transfection, the cells were collected for the Western blot analysis. CEBPB siRNA sequences were sense 5′-CGCCUGCUUUAAAUCCOUTT-3′ and antisense 5′- AUG​GAU​UUU​AAA​GGC​AGC​GTT-3'. The CEBPB negative control sequences were sense 5′-UUC​UCC​GAA​CGU​GUC​ACG​GUT​T-3′ and antisense 5′-ACG​UGA​CAC​GUU​CGG​AGA​ATT-3'.

### Microbial Community Analysis

The feces of mice in the normal group, the DSS group, and the nintedanib (100 mg/kg) group were collected and stored at −80°C. For 16S rDNA gene sequencing, fecal samples were sent to the gene and genome analysis center (Microread gene, Beijing, China) for sequencing under dry ice conditions. Primers corresponding to the bacterial 16S rRNA hypervariable region (V3–V4) were selected for amplification. The sequencing results of all samples and the statistical results of sequence data are based on sequencing reads and operational taxonomic units (OTUs).

### Statistical Analysis

All the data were analyzed using GraphPad Prism 8.4.2 (GraphPad Software, Inc., San Diego, CA) and expressed as mean ± SD. Two and multiple groups were compared using the independent sample t-test and ANOVA, respectively. *p* < 0.05 was considered statistically significant.

## Results

### Identification of CEBPB/PCK1 and CEBPB/EFNA1 Pathways Driven by Potentially Pathogenic Super-Enhancers in Inflammatory Bowel Disease

**FIGURE 1:**
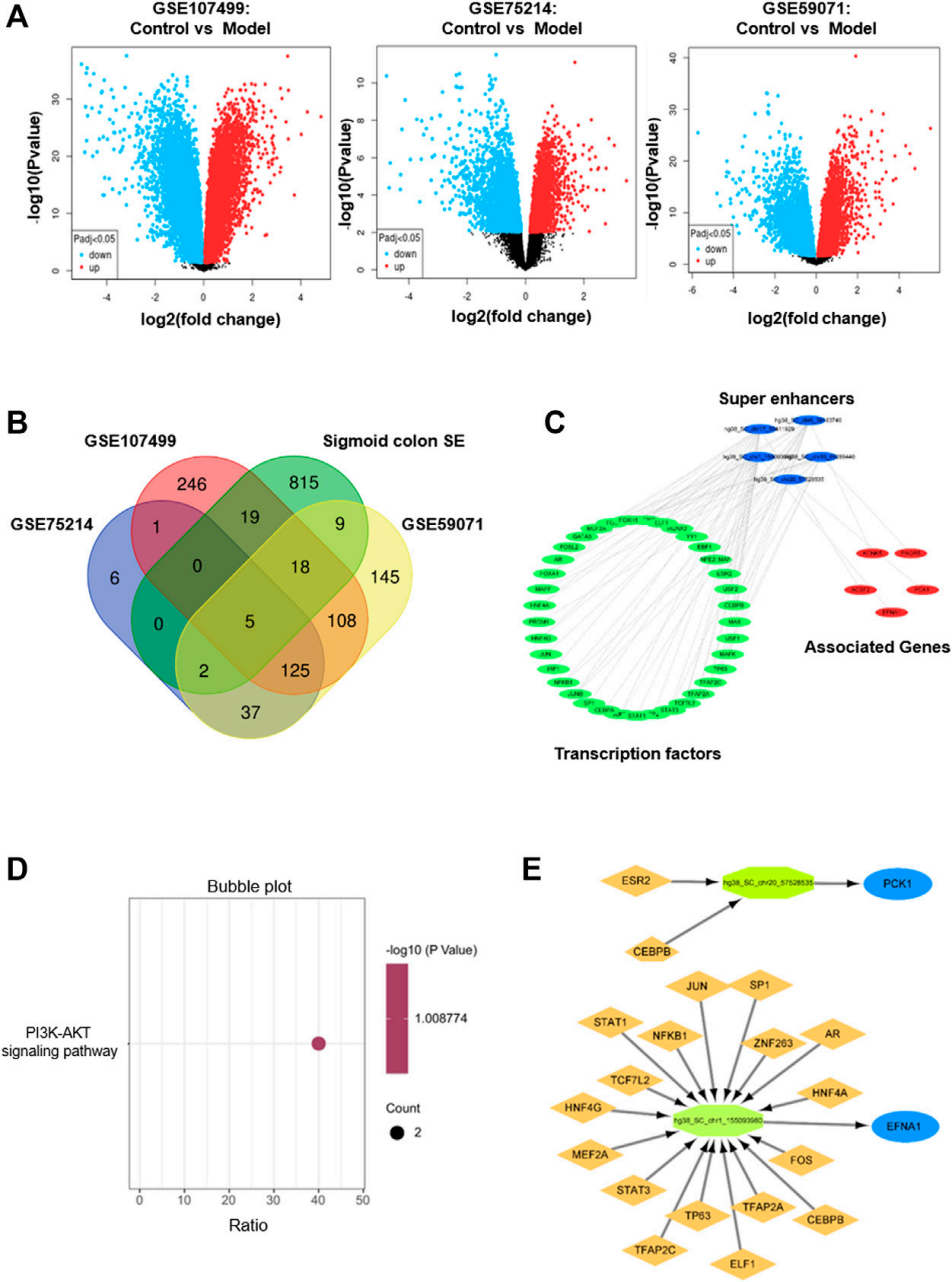
Identification of potential pathogenic super-enhancer–driver genes in IBD. **(A)** Volcano diagram of differentially expressed genes between IBD patients and normal people in datasets GSE107499, GSE75214, and GSE59071 in the GEO database. **(B)** Venn diagram formed by cross-linking of super-enhancer–associated genes and differentially expressed genes in dataset in inflammatory bowel disease. **(C)** Interaction network of TF, super-enhancer, and related genes in inflammatory bowel disease. **(D)** The KEGG pathway analysis. **(E)** TFs binding PCK1 and EFNA1 super-enhancers.

We first analyzed the GEO database (GSE107499\GSE75214\GSE59071), and the DEGs volcano is shown in Figure 1A. Super-enhancers usually positively regulate gene expression, so we analyzed the upregulated DEGs in the database. A total of 1,366 upregulated DEGs in IBD patients were found using three different GEO datasets (Table 1). 863 super-enhancer–related genes were identified in the human sigmoid colon by the SEA website. We cross-linked these super-enhancer–related genes with upregulated DEGs to predict potential super-enhancer–driven pathogenic genes in IBD. The Venn diagram shows that there are 5 gene overlaps (Figure 1B), which are considered potential super-enhancer–driven IBD pathogenic genes. The information on potential pathogenic SEs of IBD is shown in Table 2. Cytoscape software was used to construct the interaction network of transcription factors, super-enhancers, and related genes (Figure 1C). Subsequently, we performed KEGG network analysis of these five potential super-enhancer–driven pathogenic genes and found that PCK1 and EFNA1 genes were enriched in the PI3K–Akt pathway (Table 3, Figure 1D). Subsequently, we constructed the interaction networks of these two genes with super-enhancers and TFs. Surprisingly, we found that the transcription factor CEBPB existed in the relationship between the two interaction networks at the same time (Figure 1E). Therefore, we believe that CEBPB/PCK1 and CEBPB/EFNA1 may be the key pathways for the potential treatment of IBD.

**TABLE 1:** DEGs in colon tissue or mucosa of IBD from GEO datasets.

No.	Datasets	Control group	Model group	Sample number	Upregulated DEGs	Downregulated DEGs
				C/M	M vs. C	M vs. C
1	GSE107499	Human colon tissue biospy_Non-lesional	Human colon tissue biospy_lesional	44/75	825	1684
2	GSE75214	Biopsy from normal colonic mucosa of control individual	Biopsy from inflamed colonic mucosa of active CD patient	11/8	180	572
3	GSE59071	Biopsy from normal colonic mucosa of control individual	Biopsy from inflamed colonic mucosa of active UC patient	11/74	461	841

C, control group; M, model group.

**TABLE 2:** Information of potential pathogenic super-enhancers in IBD.

SEID	Loci	Length	Associated gene	Tissue type
1527529	chr17:5041192950459994	48,065	ACSF2	Sigmoid_Colon
1526949	chr1:155093980155132239	38,259	EFNA1	Sigmoid_Colon
1527572	chr20:5752853557546613	18,078	PCK1	Sigmoid_Colon
1526704	chr15:6928844669329461	41,015	PAQR5	Sigmoid_Colon
1526811	chr6:39183746–39223978	40,232	KCNK5	Sigmoid_Colon

SEID: ID of super-enhancer in SEA.

**TABLE 3:** The result of the KEGG pathway enrichment analysis.

Term	Genes	Count	Gene ratio	*p*-value (E)
PI3K–Akt signaling pathway	EFNA1\PCK1	2	40	9.8–2

### Silencing Transcription Factor CEBPB Significantly Inhibits the Expression of PCK1 and EFNA1 and Increases the Expression of Intestinal Epithelial Barrier Protein

**FIGURE 2:**
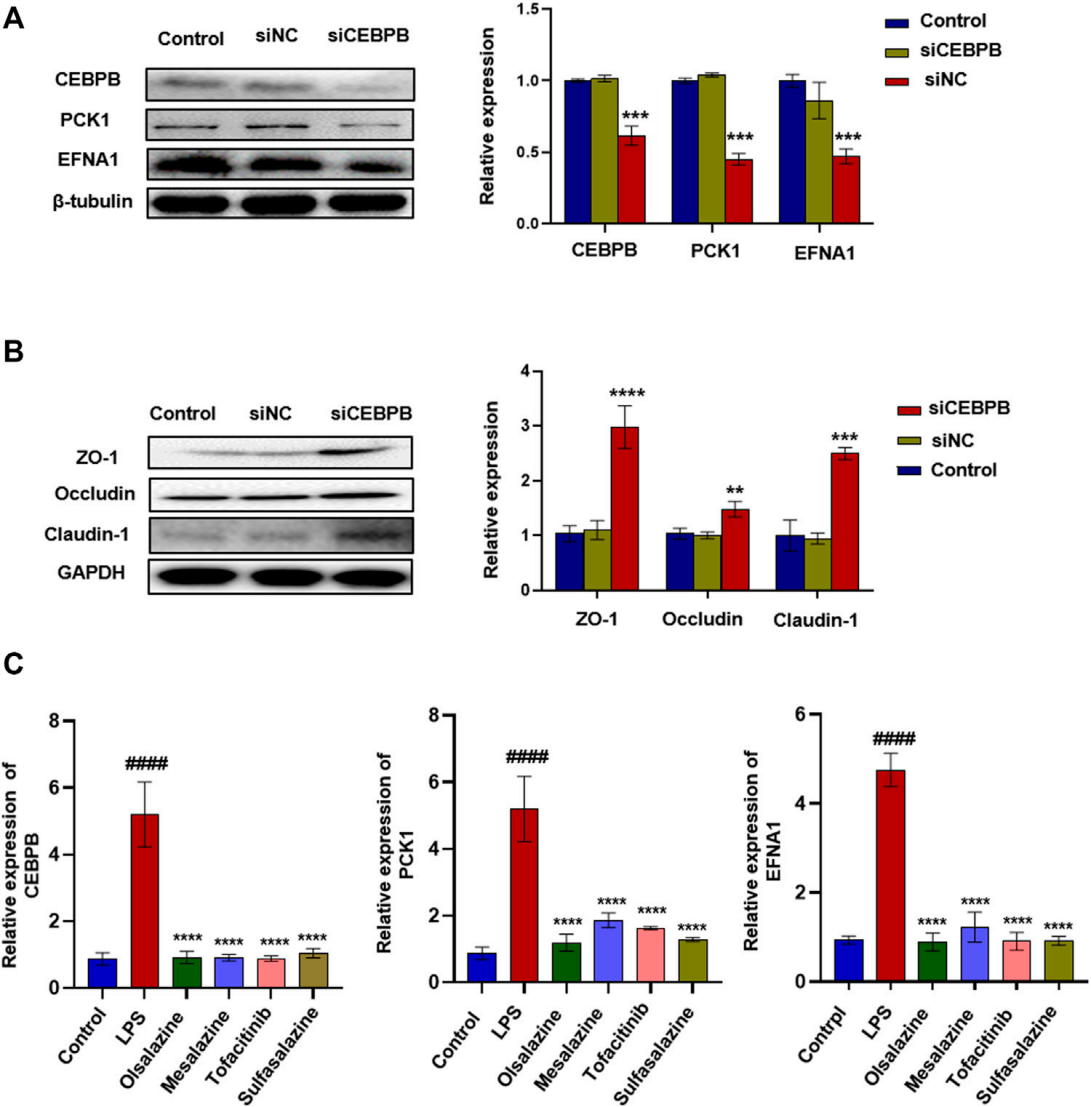
Silencing transcription factor CEBPB significantly inhibited the expression of PCK1\EFNA1 and increased the expression of intestinal epithelial barrier protein. The protein expression of super-enhancer–associated gene PCK1\EFNA1 **(A)** and intestinal barrier proteins ZO-1, occludin, and claudin-1 **(B)** after transfection of siCEBPB. Data are presented as the mean ± SD (*n* = 3). ^*^ *p* < 0.01 and ^**^ *p* < 0.05 significantly different from the control group. **(C)** Inhibitory effect of IBD marketed drugs (olsalazine, mesalazine, tofacitinib, and sulfasalazine) on CEBPB, PCK1, and CEBPB mRNA. Data are presented as the mean ± SD (*n* = 3). ^*####*^ *p* < 0.001 significantly different from the control group; ^*^ *p* < 0.1, ^***^ *p* < 0.001 and ^****^ *p* < 0.0001, significantly different from LPS groups.

To verify whether CEBPB regulates PCK1 and EFNA1 to improve colitis, we designed the interfering RNA of CEBPB. As shown in Figure 2A, after silencing the transcription factor CEBPB, the protein expression levels of PCK1 and EFNA1 decreased significantly. This indicates that PCK1 and EFNA1 are regulated by the transcription factor CEBPB. At the same time, silencing the transcription factor CEBPB also found that the intestinal barrier proteins ZO-1, occludin, and claudin-1 increased (Figure 2B), indicating that inhibiting CEBPB can restore the damaged intestinal barrier proteins. The aforementioned results indicate that PCK1 and EFNA1 pathways regulated by CEBPB may participate in the pathogenesis of IBD through epithelial barrier proteins. Subsequently, we detected the mRNA levels of CEBPB, PCK1, and EFNA1 in LPS-stimulated Caco-2 cells after being treated with the marketed drugs commonly used for the treatment of IBD (sulfasalazine, mesalazine, olsalazine, and tofacitinib). The results showed that LPS significantly promoted the expression of CEBPB, PCK1, and EFNA1 mRNA in Caco-2 cells, while sulfasalazine, mesalazine, olsalazine, and tofacitinib significantly inhibited the expression of CEBPB, PCK1, and EFNA1 mRNA stimulated by LPS (Figure 2C).

### Nintedanib Significantly Inhibits the CEBPB/PCK1 and CEBPB/EFNA1 Pathways

**FIGURE 3:**
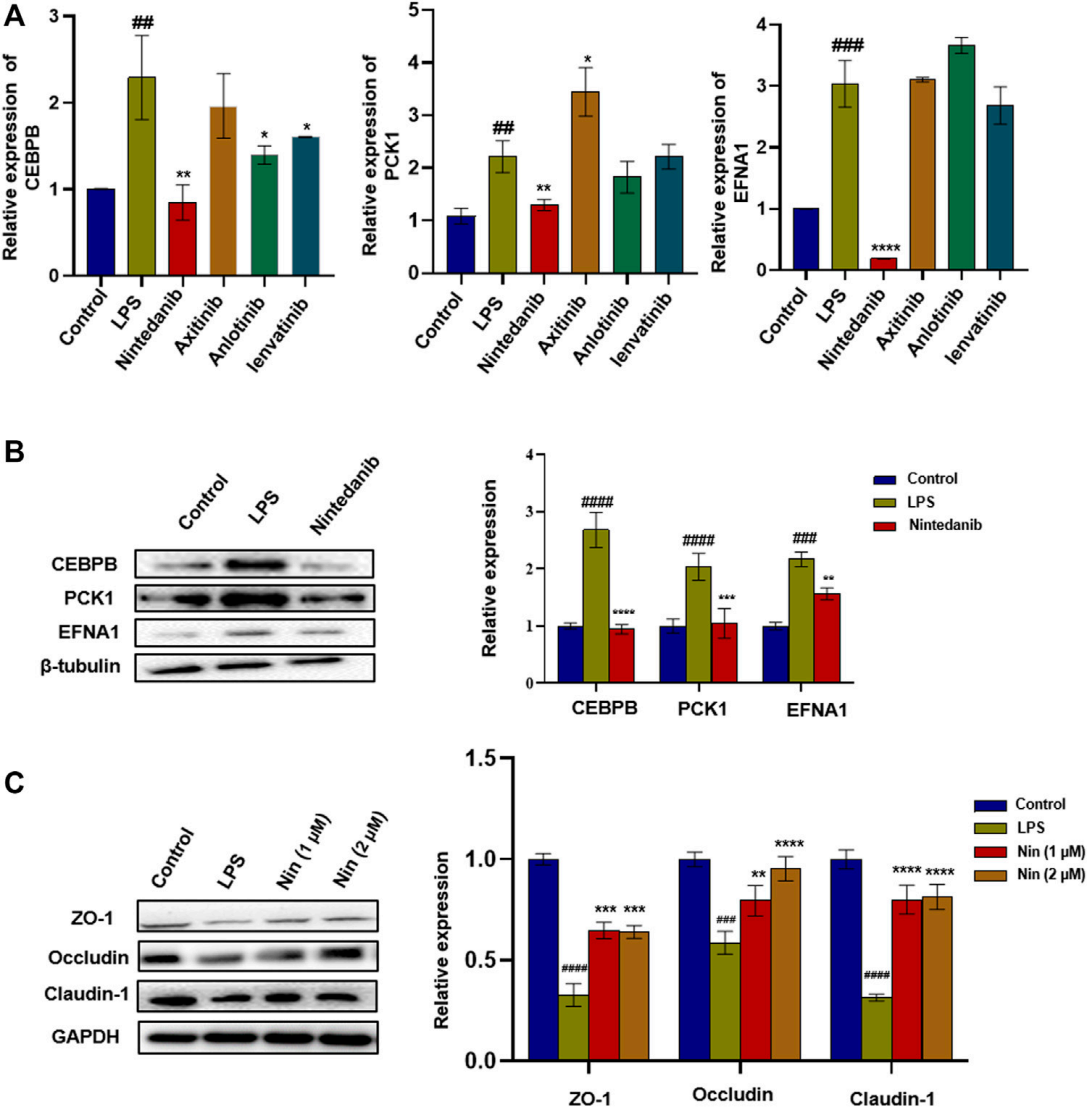
Screening of tyrosine kinase inhibitors and the effect of nintedanib on barrier protein in Caco-2 cells. **(A)** Screening of tyrosine kinase inhibitors in LPS-induced Caco-2 cells. **(B)** Nintedanib significantly inhibited the protein expression levels of CEBPB, PCK1, and EFNA1. **(C)** Nintedanib improves the injury of intestinal epithelial barrier protein *in vitro*. Data are presented as the mean ± SD (*n* = 3). ^*#*^ *p* < 0.05, ^*##*^ *p* < 0.01, ^*###*^ *p* < 0.001 and ^*####*^ *p* < 0.0001 significantly different from the control group; ^*^ *p* < 0.05, ^**^ *p* < 0.01, ^***^ *p* < 0.001, and ^****^ *p* < 0.0001, significantly different from LPS groups.

The aforementioned four marketed drugs have different degrees of side effects, so it is necessary to develop new safe and effective drugs. Some small-molecule drugs of tyrosine kinase inhibitors (such as nintedanib and anlotinib) have been proved to have certain anti-inflammatory effects (Wollin et al., 2014). We wanted to determine the efficacy of tyrosine kinase inhibitors such as anlotinib, lenvatinib, axitinib, and nintedanib in treating IBD. As shown in Figure 3A, compared with the normal group, the mRNA levels of CEBPB, PCK1, and EFNA1 in the LPS group increased significantly. Similar to tofacitinib, sulfasalazine, oxalazine, and mesalazine, mRNA levels of CEBPB, PCK1, and EFNA1 in the nintedanib group were significantly reduced after administration, while the mRNA levels in anlotinib, lenvatinib, and axitinib groups did not change significantly after administration. After treating LPS-induced Caco-2 cells with nintedanib, it was found that the nintedanib group could significantly reduce the protein expression levels of CEBPB, PCK1, and EFNA1. At the same time, nintedanib can repair the damage to intestinal barrier protein caused by LPS (Figure 3B,C). These results suggest that nintedanib may alleviate IBD by inhibiting CEBPB/PCK1 and CEBPB/EFNA1 pathways.

### Nintedanib Improves DSS-Induced Experimental Colitis in Mice

**FIGURE 4:**
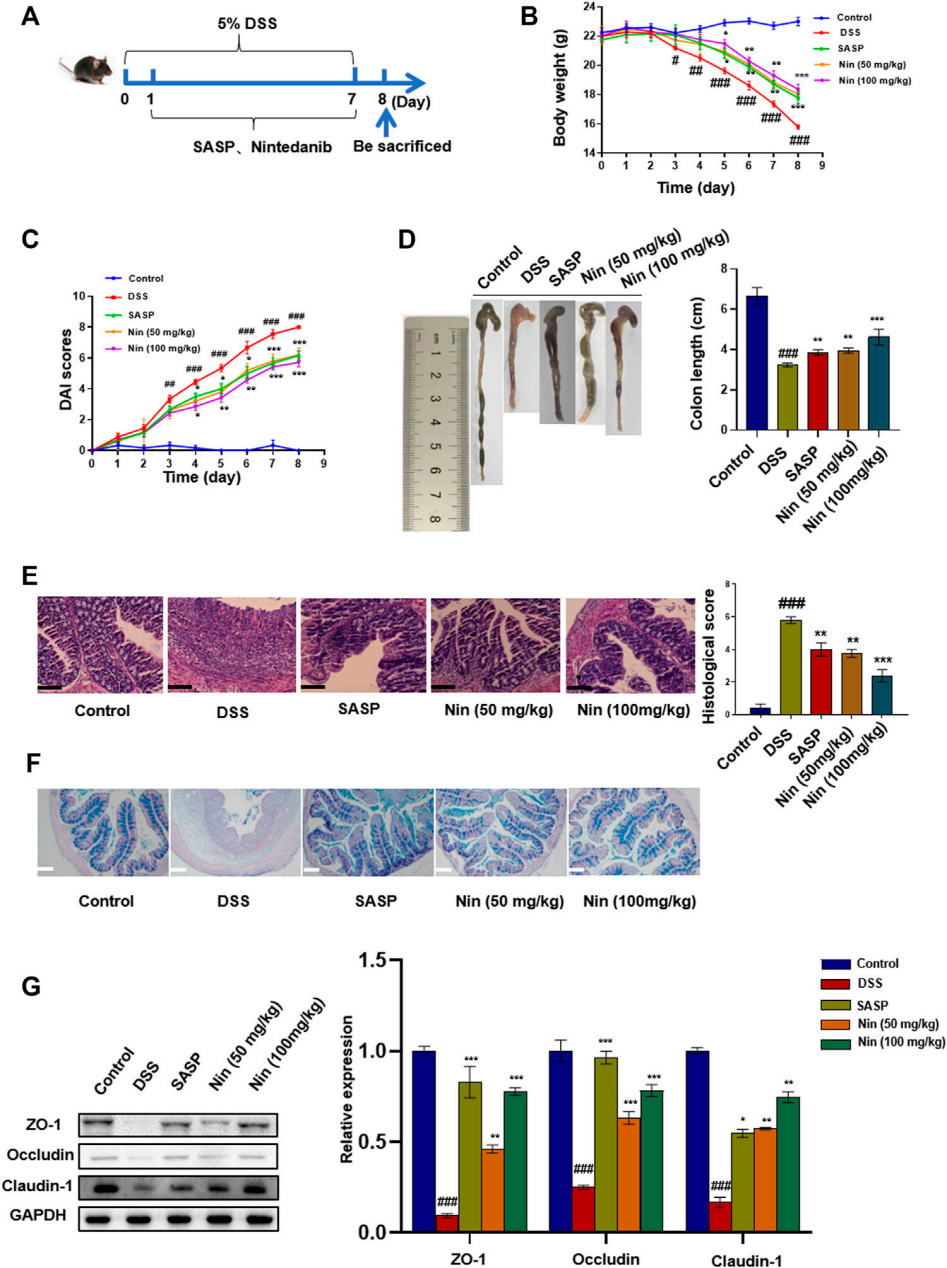
Effect of nintedanib on DSS-induced experimental colitis in mice. **(A)** 5% DSS-induced experimental colitis in mice. Effects of nintedanib on weight change **(B)**, the DAI score **(C)**, and colon length change **(D)** in experimental colitis mice. **(E)** Histological sections of colonic tissue stained with hematoxylin and eosin are shown using a microscope. Scale bars represent 50 μm. **(F)** Representative images of Alcian blue-stained inner mucus layer of colonic sections. Scale bars represent 50 μm. **(G)** The effect of nintedanib on the expression of intestinal epithelial barrier proteins ZO-1, occludin, and claudin-1. Data are presented as the mean ± SD (*n* = 5). ^*##*^ *p* < 0.01, ^*###*^ *p* < 0.001, significantly different from the control group; ^*^ *p* < 0.05, ^**^ *p* < 0.01, ^***^ *p* < 0.001, significantly different from DSS groups.

To further verify the therapeutic effect of nintedanib on colitis, we used 5% DSS to induce colitis in mice. As shown in Figure 4A, the murine experimental colitis animal model was established. Compared with the control group, DSS-treated mice significantly reduced body weight, increased DAI, and shortened colon weight length (Figure 4B,C,D). Significant tissue damage was found in the histological examination of the colon of DSS-treated mice, with a high microdamage score (Figure 4E). The positive control SASP (100 mg/kg) and nintedanib (50, 100 mg/kg) could significantly recover the experimental colitis-related symptoms of mice caused by DSS, such as weight loss, increased DAI, shortened colon length, and colonic tissue injury.

Alcian blue staining was used to observe the expression of intestinal mucins. Intestinal mucin is stained blue by Alcian blue, and nuclei are stained red by nuclear fast red fuel. As shown in Figure 4F, DSS caused damage to the intestinal mucosa, with a significant decrease in the mucin expression. The intestinal mucosal injury caused by DSS was alleviated by intragastric administration [SASP, nintedanib (50, 100 mg/kg)].

We conducted the Western blot test on colonic tissue and found that the intestinal barrier protein in the DSS group was seriously damaged. The positive drug group and low- and high-dose groups (50 and 100 mg/kg) of nintedanib could significantly recover the damaged intestinal barrier protein, and the high-dose group of nintedanib had a better effect (Figure 4G).

### Nintedanib Improves DSS-Induced Experimental Colitis in Mice by Inhibiting CEBPB/PCK1 and CEBPB/EFNA1 Pathways

**FIGURE 5:**
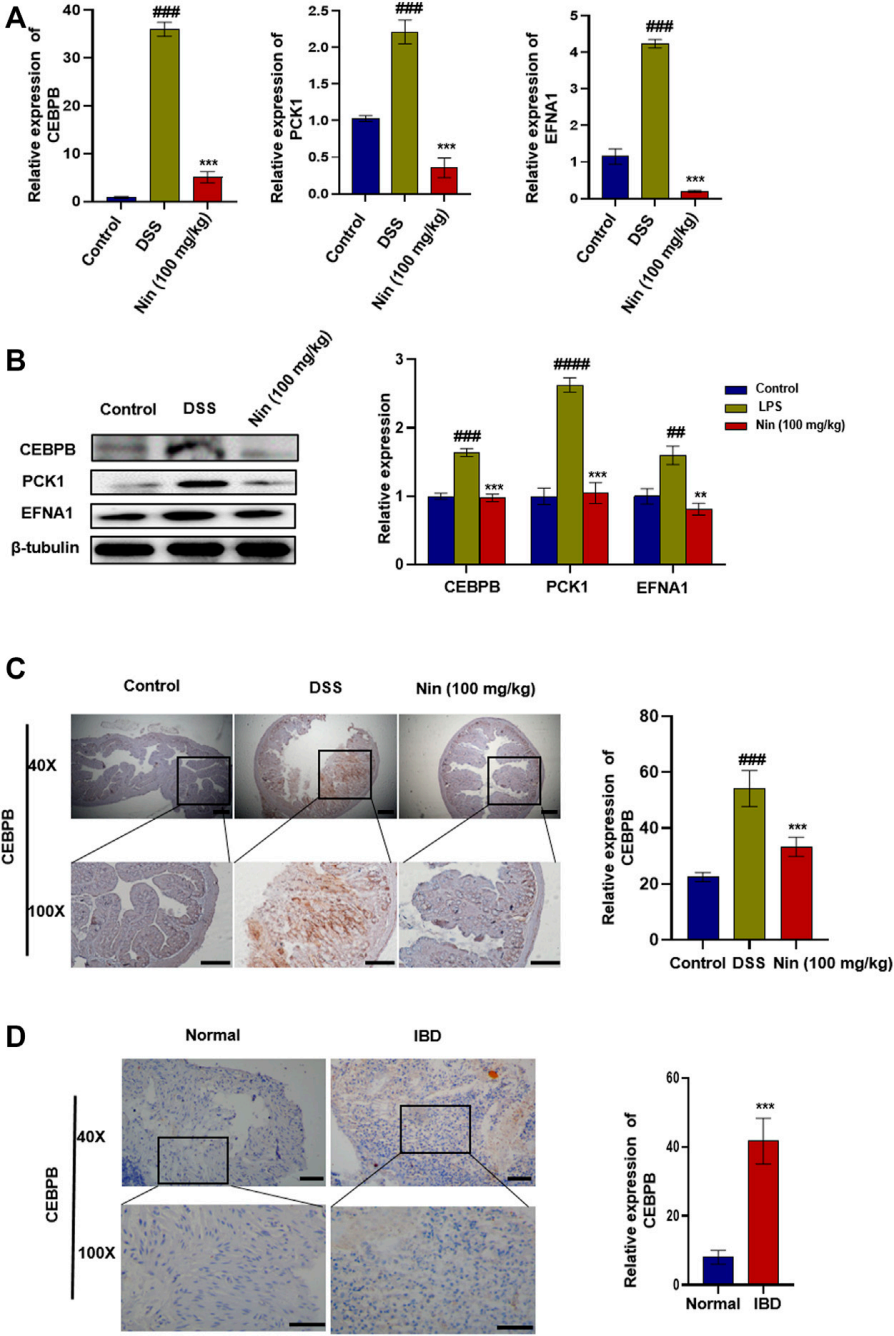
Inhibition of CEBPB/PCK1 and CEBPB/EFNA1 pathways by nintedanib *in vivo*. **(A)** Effect of nintedanib on the level of CEBPB, PCK1, and EFNA1 mRNA in the colon tissue. **(B)** Effect of nintedanib on the expression of CEBPB, PCK1, and EFNA1 proteins in the colon tissue. The IHC staining analysis of CEBPB in mouse **(C)** and human **(D)** colon tissues. Scale bars represent 50 μm. Data are presented as the mean ± SD (*n* = 5). ^*#*^ *p* < 0.05, ^*##*^ *p* < 0.01, ^*###*^ *p* < 0.001 and ^*####*^ *p* < 0.0001, significantly different from the control group; ^*^ *p* < 0.05, ^**^ *p* < 0.01, ^***^ *p* < 0.001 and ^****^ *p* < 0.0001, significantly different from DSS groups.

As shown in Figure 5A,B, the mRNA and protein expression of CEBPB, PCK1, and EFNA1 in the DSS group increased significantly, and the mRNA and protein expression of CEBPB, PCK1, and EFNA1 decreased significantly after nintedanib administration. IHC results showed that CEBPB increased significantly after DSS treatment, while CEBPB immunostaining decreased significantly after nintedanib treatment (Figure 5C). These results suggested that nintedanib may inhibit DSS-induced experimental colitis in mice by inhibiting CEBPB/PCK1 and CEBPB/EFNA1 pathways. IHC of tissue sections from healthy individuals and IBD patients similarly demonstrated a significant increase in CEBPB in tissue sections from IBD patients (Figure 5D). The patient information is shown in Table 4.

**TABLE 4:** Basic information of patients.

Pathology number	Gender	Age
1414251	Female	58
1314397	Male	37
1730153	Female	63
1704203	Female	49
1310072	Male	35
1702166	Male	54

### Effects of Nintedanib on Gut Microbiota of Experimental Colitis Mice

**FIGURE 6:**
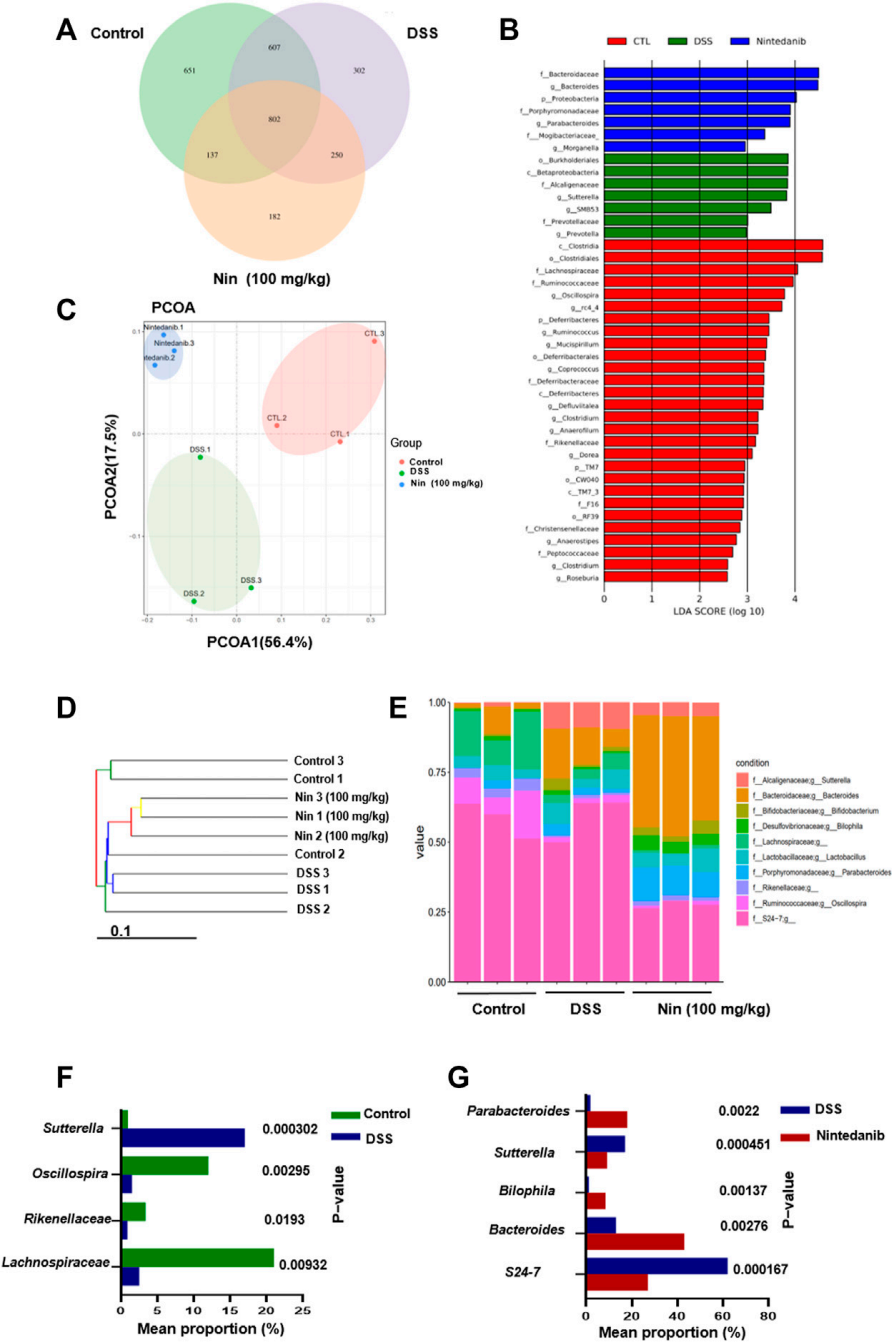
Nintedanib regulates the gut microbiota composition of DSS-induced experimental colitis. **(A)** The Venn diagram showing the unique and shared OTUs from control, DSS, and nintedanib groups. **(B)** The difference of OTUs among the control group, the DSS group, and the nintedanib group was determined by linear discriminant analysis combined with effect size (lefse). **(C)** PCOA of gut microbiota communities based on the OTU level. **(D)** The clustering analysis of the evolution of the gut microbiotas between control, DSS, and nintedanib groups. Based on the distance matrix, the gut microbiota tree was constructed using the UPGMA (unweighted pair group method with arithmetic mean) clustering method for analysis. **(E)** Histograms of relative abundance of grouped species at the genus level. **(F)** Gut microbiota differentially expressed at genus level between control and DSS groups. **(G)** Gut microbiota differentially expressed at genus level between DSS and nintedanib groups.

Since the gut microbiota is an important factor contributing to IBD, we utilized bacterial 16S rRNA sequencing to investigate the modulation of gut microbiota composition by nintedanib in mice. As shown in Figure 6A, we first used the Venn diagram to visualize the results of OTU cluster analysis between different groups. In total, 2931 OTUs were obtained from the feces of control, DSS-, and nintedanib-treated mice, 802 of which were shared by all three groups. The number of unique OTUs in the control, DSS, and nintedanib groups was 651, 302, and 182, respectively. Next, as shown in Figure 6B, we used linear discriminant analysis effect size (lefse) to identify differentially expressed OTUs between the feces of different groups of mice. Principal coordinate analysis (PCOA) was used to analyze the community structure of the mouse gut microbiota in the three groups (Figure 6C). On the PCOA plot, each symbol represents one gut microbiota. The more similar the abundance and composition of species, the closer the distance between samples. The results showed that the gut microbiota was significantly different between the different groups. The relationship between the gut microbiota from the three groups of fecal samples was investigated using a gut microbiota tree generated by the UPGMA algorithm. The dendrogram (Figure 6D) described and compared the similarity and difference relationship between the three samples intuitively through the dendritic structure. Figure 6E showed that the composition of the gut microbiota in different groups was significantly different at the genus level. After DSS treatment, the relative abundances of *Lachnospiraceae*, *Rikenellaceae*, and *Oscillospira* significantly decreased and that of *Sutterella* significantly increased compared with those of the control group (Figure 6F). After nintedanib administration, the gut microbiota of the mice was significantly altered compared with the DSS group (Figure 6G). The relative abundances of *Sutterella* and *S24-7* decreased, and those of *Bacteroides*, *Bilophila*, and *Parabacteroides* increased significantly, with *Bacteroides* becoming the dominant genus. Collectively, the gut microbiota of the control, DSS, and nintedanib groups were significantly different, and nintedanib significantly changed the composition of the intestinal microbiota in mice with DSS-induced experimental colitis.

## Discussion

IBD is an autoimmune disease. The destruction of the intestinal epithelial barrier and the increase of mucosal permeability activate the immune response, resulting in tissue injury, pathological changes, and clinical manifestations of IBD (Wendelsdorf et al., 2010). Super-enhancers regulate gene transcription by aggregating TFs, such as abnormal transcription driven by super-enhancers in most cancer cells (Sengupta and George, 2017). It has been reported that the genetic changes of super-enhancers can cause gene transcription imbalance (Sengupta and George, 2017). At present, it has been found that SNPs often appear in the super-enhancer region in a variety of autoimmune diseases such as systemic lupus erythematosus (Vahedi et al., 2015). In one study, 13 of the 76 SNPs in type 1 diabetes patients were located in the super-enhancers region (Hnisz et al., 2013). Because super-enhancers are closely related to the occurrence and development of autoimmune diseases, we have reason to believe that super-enhancers and their driving genes are related to the pathogenesis of IBD. This study studied the role of TFs, super-enhancers and their regulatory genes in IBD, and screened some existing drugs in the market. The results show that CEBPB/PCK1 and CEBPB/EFNA1 pathways can be used to study the pathogenesis of IBD, and nintedanib can effectively alleviate IBD.

Super-enhancer is an ultralong cis-acting element that controls gene transcription (Cheng et al., 2016). It controls gene expression by enriching transcription factors and binding to specific DNA sequences. We analyzed the data in the GEO database, found the upregulated DEGs (PCK1 and EFNA1) in IBD patients compared with normal people, and constructed CEBPB/PCK1 and CEBPB/EFNA1 pathways composed of TFs, super-enhancers, and their related genes. Such pathways are considered to play an important role in the occurrence of IBD.

The function of super-enhancers is reflected by their associated genes (Li et al., 2021a). KEGG pathway gene enrichment analysis is helpful to find out the possible roles of these super-enhancer–associated genes in the development and progression of IBD. Interestingly, the PI3K–Akt signaling pathway was the most important one. It is well known that PI3K–Akt has a close relationship with the occurrence and development of intestinal inflammation (Gao et al., 2021).

CEBPB is an important transcription factor that belongs to the CCAAT enhancer-binding proteins family. It is mainly involved in important life activities such as cell proliferation and differentiation, tumorigenesis and apoptosis, body inflammatory response, and so on through the regulation of gene transcription of target cells (Ramji and Foka, 2002). PCK1 and EFNA1 have been found to be involved in important life activities such as cell proliferation and apoptosis, tumor angiogenesis, malignant cell events, and invasiveness (Sadasivam et al., 2014), and their expression is upregulated in a variety of tumors [such as colorectal cancer (Shi et al., 2012)]. However, whether there is a link between transcription factors and super-enhancer–driven genes is still unknown. Therefore, we designed the simulant of CEBPB to inhibit the activity of transcription factors. Surprisingly, after silencing transcription factor CEBPB, the expression of super-enhancer–related genes decreased, which proved that transcription factor CEBPB affected the expression of super-enhancer–driving genes PCK1 and EFNA1.

The destruction of intestinal epithelial barrier protein is an important manifestation of colitis. The destruction of intestinal epithelial barrier function will lead to the immune response of the body and produce the clinicopathological manifestations of IBD (Su et al., 2009; Novak and Mollen, 2015). ZO-1 (Furuse et al., 1994), occludin (Furuse et al., 1998), and claudin-1 are intestinal epithelial barrier proteins, which are very important for the stability of epithelial barrier function. Previous studies have shown that geniposide and diosmetin can improve colitis by significantly increasing the damaged intestinal barrier protein (Xu et al., 2017; Li et al., 2021b). In the experiment, we found that the expression of related barrier proteins ZO-1, occludin, and claudin-1 increased significantly after silencing the transcription factor CEBPB. This indicates that CEBPB/PCK1 and CEBPB/EFNA1 pathways affect the expression of intestinal barrier protein. Sulfasalazine, mesalazine, olsalazine, and tofacitinib are the first-line drugs in the treatment of IBD (Tursi et al., 2008; Song et al., 2017; Sayoc-Becerra et al., 2020). In order to further prove the role of CEBPB/PCK1 and CEBPB/EFNA1 pathways in IBD, we selected positive drugs for verification. The results were as expected, the listed drugs could significantly inhibit the mRNA expression levels of CEBPB, PCK1, and EFNA1.

As we all know, the safety of listed drugs is known and the development of new indications can greatly save development time and cost. Tyrosine kinase inhibitors are most often developed as cancer targets (van Vollenhoven et al., 2012; Huang et al., 2020). Later, it was found that some of them have anti-inflammatory properties (Roskoski, 2016; McInnes and Schett, 2017). Previous studies by us and others have found that some tyrosine kinase inhibitors have anti-inflammatory effects in pulmonary fibrosis, but none have investigated this in enteritis (Varone et al., 2018; Ruan et al., 2020). Therefore, we used these two pathways to screen some existing tyrosine kinase inhibitors (anlotinib, lenvatinib, axitinib, and nintedanib) to study whether they have a certain role in the treatment of IBD. Our data showed that nintedanib can significantly inhibit the mRNA expression levels of CEBPB, PCK1, and EFNA1 at the same time. Therefore, we chose nintedanib for further verification. Consistent with the aforementioned results, in Caco-2 cells, nintedanib could significantly inhibit the protein expression levels of CEBPB, PCK1, and EFNA1, and aid the destruction of intestinal barrier protein induced by LPS.

Studies have shown that gut microbiota plays a key role in the pathogenesis of IBD (Hernández-Chirlaque et al., 2016). The study by Shuang Jin et al. found that significant changes in gut microbiota can lead to IBD (Jin et al., 2017). Here, we studied the effects of DSS and nintedanib on the changes in gut microbiota in mice. We analyzed the effect of nintedanib on the composition of the gut microbial community. At the genus level, there are fundamental changes in the compositional structure of the gut microbial community. In the nintedanib group, the *Bacteroidetes* substitution *S24-7* became the most abundant strain. *Bacteroidetes* is an important keystone bacterium (Wilson et al., 1997; Franks et al., 1998; Hayashi et al., 2002), which has a significant impact on human health, especially in sugar fermentation and polysaccharide (Xu et al., 2003). *Sutterella* is commonly associated with human diseases, such as autism (Dan et al., 2020) and inflammatory bowel disease (Kaakoush, 2020), but the health effects of these bacteria remain unclear and are considered harmful in several studies (Cai et al., 2019). Nevertheless, there is no doubt that nintedanib reversed the trend of increased *sutterella* abundance compared with the DSS group. *Parabacteroides*, one of the core microbiota in humans, is a potential, novel antimetabolic syndrome probiotic, and here, we could find a significant increase in the abundance of *Parabacteroides* (Kai Wang et al., 2019). The results showed that the composition of gut microbiota in mice with experimental colitis was improved by nintedanib at the genus level, which may account for the therapeutic efficacy of nintedanib in experimental colitis.

## Conclusion

In summary, this study shows that inhibition of super-enhancer–driven CEBPB/PCK1 and CEBPB/EFNA1 signaling pathways can reduce LPS-induced barrier dysfunction *in vitro* and improve DSS-induced experimental colitis *in vivo*. Nintedanib has a certain role in the treatment of IBD. However, there are still many deficiencies in this study. For example, there are few studies on the transcriptional enhancement effect of super-enhancers (SC-CHR20-57528535 and SC-CHR1-155093980) on the transcription factor CEBPB, which is the focus of our next study. In conclusion, this study provides a new idea for the pathogenesis of enteritis and the exploration of candidate drugs.

## Data Availability

The datasets presented in this study can be found in online repositories. The names of the repository/repositories and accession number(s) can be found in the article/Supplementary Material.
